# Combined effect of nutritional inflammation as well as depression on mortality in middle-aged and elderly people with osteoporosis and osteopenia

**DOI:** 10.1371/journal.pone.0335272

**Published:** 2025-11-03

**Authors:** Yazhou Liu, Zhe Yang, Yuhao Li, Xiaodong Yang

**Affiliations:** 1 Department of Orthopedics, Dalian Medical University, Dalian, China; 2 Department of Orthopedics, Dandong Central Hospital, Dalian Medical University, Dandong, China; 3 Department of Suzhou University, Suzhou, China; 4 Department of Orthopedics, Dandong Central Hospital, China Medical University, Dandong, China; NUST: National University of Sciences and Technology, PAKISTAN

## Abstract

**Background:**

Inflammation, nutritional status, and depression interact complexly, impacting health outcomes. This study investigates their associations with all-cause and cardiovascular mortality in middle-aged and elderly individuals with osteoporosis.

**Methods:**

Using NHANES data from 2007 to 2023, the study applied Cox regression models and restricted cubic spline plots to assess the effects of ALI (Advanced Lung Cancer Inflammation Index) and PHQ-9 scores on mortality outcomes in osteoporosis patients. Subgroup, threshold, and mediation analyses were also conducted.

**Results:**

The study included 862 cases of all-cause mortality and 211 cardiovascular deaths. Higher ALI was associated with reduced mortality risk, while higher PHQ-9 scores indicated increased mortality risk. Combined analysis showed that osteoporosis patients with high ALI and no depressive symptoms had the lowest mortality risk. Restricted cubic spline and threshold analyses revealed a linear negative correlation between ALI and mortality risk and a nonlinear positive correlation between PHQ-9 scores and mortality risk. Subgroup analysis showed gender, physical activity, and sleep status influenced the interaction between ALI/PHQ-9 and mortality risk. Causal mediation analysis with bootstrapping found that ALI mediated 3.9% of the effect of osteoporosis on all-cause mortality and 5.6% on cardiovascular mortality, while PHQ-9 scores mediated 6.6% of cardiovascular mortality.

**Conclusion:**

A significant negative correlation exists between ALI and mortality risk in osteoporosis patients, while PHQ-9 scores correlate positively. Favorable nutrition and inflammation, coupled with the absence of depression, help reduce mortality risks.

## Introduction

Osteoporosis (OP) is a systemic metabolic bone disorder characterized by a decrease in bone mineral density and the deterioration of bone microarchitecture [1,2]. It significantly elevates the risk of fractures, compromises bone strength, and may lead to severe complications, including pain, deformities, and functional impairments. These consequences not only impose a substantial burden on the patient’s health but also considerably affect their quality of life. The incidence and mortality rates of fractures attributable to osteoporosis are notably high, with a lifetime fracture risk of 40–50% for women and 13–22% for men [3]. Redlich, Godde, and colleagues have noted that the long-term survival of patients with osteoporosis is influenced by a range of factors, including tissue damage, the extent of inflammation, and the individual’s mental health status [4,5]. Despite notable advancements in treatment strategies, patients with osteoporosis continue to encounter persistent physiological and psychological challenges, which can significantly affect their long-term health outcomes [6]. Inflammation and malnutrition associated with osteoporosis can lead to alterations in immune function and metabolic processes, thereby impacting the patient’s survival prognosis. Consequently, it is essential to identify and intervene in modifiable factors that could enhance the long-term prognosis of osteoporosis patients.

In recent years, an increasing body of research has demonstrated that various nutrition- and inflammation-related factors are closely linked to the onset and progression of osteoporosis [7]. Moreover, malnutrition is recognized as one of the key risk factors for osteoporosis and is significantly associated with an elevated risk of fractures [8]. Notably, malnutrition and inflammation often interact in a synergistic manner. Inflammatory responses can accelerate the hydrolysis and degradation of muscle proteins while impairing repair mechanisms, resulting in a reduction in muscle mass. Simultaneously, malnutrition can compromise immune function, thereby heightening susceptibility to infections and aggravating the inflammatory response [9,10].

In addition to the effects of inflammation and malnutrition, depression has emerged as a critical factor influencing the onset, progression, and survival outcomes of osteoporosis [11,12]. Not only is depression associated with an increased incidence of osteoporosis, but it is also strongly correlated with a decline in the quality of life among osteoporosis patients [13,14]. The relationship between depression and inflammation is intricate and multifaceted [15,16], with some epidemiological studies indicating that depression may influence inflammatory processes, while others suggest that inflammation could serve as a precursor to depression [17]. Moreover, some researchers propose a bidirectional relationship between depression and inflammation [18]. Despite this, the evidence regarding the association between inflammation, depressive symptoms, and mortality remains limited, particularly in the context of osteoporosis.

Given the complex interplay between nutritional status and chronic inflammation in the pathophysiology of osteoporosis, a composite index that simultaneously captures both critical domains could offer a more holistic prognostic assessment than single biomarkers. The Advanced Lung Cancer Inflammation Index (ALI), calculated from Body Mass Index (BMI), serum albumin, and the Neutrophil-to-Lymphocyte Ratio (NLR), is one such promising biomarker. Although originally developed in oncology [19], its components are all individually established as key factors in bone metabolism. Specifically, BMI and serum albumin are well-known indicators of nutritional status, a critical determinant of bone health [20,21], while the NLR is a widely accepted marker of systemic inflammatory response, which is known to accelerate bone loss [22]. We therefore hypothesized that the ALI, by holistically integrating these crucial nutritional and inflammatory signals into a single, objective score, could serve as a powerful prognostic marker for mortality in patients with osteoporosis.

This study aims to validate the utility of the ALI as a prognostic inflammation-nutritional index and assess the impact of systemic depressive conditions on the survival of osteoporosis patients. Furthermore, we will evaluate the combined effect of inflammation-nutritional status and depression on mortality risk, with the goal of providing new insights into the long-term prognosis of osteoporosis patients and identifying potential intervention strategies to enhance their quality of life and survival outcomes.

## Methods

### 2.1 Study design and data collection

This study employed a retrospective cohort design using data from the National Health and Nutrition Examination Survey (NHANES). Data were drawn from 8 consecutive cycles, spanning from 2007–2008–2022–2023. NHANES is a nationally representative, stratified, multistage probability sampling survey managed by the National Center for Health Statistics (NCHS) [23]. Written informed consent was obtained from all participants, and the study received approval from the NCHS Institutional Review Board (IRB).

### 2.2. Study population

The study population comprised middle-aged and elderly individuals diagnosed with osteoporosis or osteopenia, as well as a control group of the general population. The exclusion criteria for the study were as follows: 1) individuals under the age of 40 with no sample weight data; 2) lack of bone mineral density (BMD) measurements; 3) lack of data on serum albumin levels, neutrophil count, lymphocyte count, BMI, and depression questionnaire responses; 4) lack of mortality data; 5) absence of covariate data.

### 2.3 Assessment of osteoporosis/osteopenia, ali, and depressive symptoms

Osteoporosis is usually diagnosed by dual-energy X-ray absorptiometry (DXA). Based on BMD measurements of the total femur (TF), femoral neck (FN), and lumbar spine (LS), participants were categorized into three groups: a normal group, an osteopenia group, and an osteoporosis group. The diagnostic criteria for osteopenia and osteoporosis were referred to the method proposed by Looker et al [24,25]. Taking male and female participants between 18 and 25 years of age, their mean BMD values were used as a reference standard. According to the World Health Organization (WHO) criteria: 1) T-values ≤ −2.5 were diagnosed as osteoporosis; 2) T-values ≥ −1.0 were considered as normal BMD; and 3) between −2.5 and −1.0 were considered as osteopenia. Therefore, the final cohort of this study included individuals diagnosed with osteoporosis or osteopenia.

The ALI was calculated as ALI = BMI × Alb/ NLR [19]. The Patient Health Questionnaire-9 (PHQ-9) is employed to diagnose and assess the severity of depressive symptoms in OP patients. The PHQ-9 consists of nine items, each scored from 0 to 3, resulting in a total score ranging from 0 to 27. Higher scores indicate greater severity of depressive symptoms. Based on the PHQ-9 scores, patients are classified into three categories: no depression (0–4 points), mild depression (5–9 points), and moderate to severe depression (≥10 points). Additionally, based on extensive studies on the validity of the PHQ-9, participants with a score ≥10 are considered to have potentially clinically significant depression.

### 2.4. Ascertainment of mortality

To assess mortality in the follow-up population, we used National Death Index (NDI) mortality data as of December 31, 2019, which is linked to the NHANES database and publicly available. To enhance the robustness of the analysis, we used a propensity score matching algorithm.

### 2.5. Covariates

This study comprehensively includes independent risk factor covariates associated with osteoporosis and osteopenia, all derived from existing literature. The specific covariates encompass age, gender, race, income-to-poverty ratio, education level, self-reported history of diabetes, self-reported history of hypertension, smoking status, alcohol consumption, physical activity level, and sleep disorders, with the aim of minimizing confounding bias. Trained interviewers utilized a computer-assisted personal interview (CAPI) system to conduct household and sampling surveys, collecting demographic data such as age, gender, race, income-to-poverty ratio, and education level. Details of the relevant definitions are shown in S1 Table in S1 File.

### 2.6. Statistical analysis

To ensure the national representativeness of the sample, we adhered to the NHANES weighting guidelines (https://www.cdc.gov/nchs/nhanes/tutorials/weighting.aspx) and applied the Mobile Examination Center (MEC) weights during the sampling process. Receiver operating characteristic (ROC) curves were employed to identify the most effective nutritional and inflammatory biomarkers for ALI within the NHANES dataset. The baseline characteristics of individuals with varying degrees of ALI and depressive symptoms were further examined. Continuous variables were expressed as weighted means with standard errors, while categorical variables were presented as frequencies and weighted proportions.

To examine the association of ALI and depressive symptoms with all-cause and cardiovascular mortality, we constructed a series of multivariable Cox proportional hazards regression models. This hierarchical approach was designed to systematically control for potential confounding variables. We developed three distinct models: Model 1: This was a crude model with no covariate adjustment. Model 2: This model was minimally adjusted for core demographic characteristics, including age (as a continuous variable), sex (male/female), and race/ethnicity. Model 3: This was our fully adjusted model, which included all covariates from Model 2 plus a comprehensive set of potential confounders selected based on established literature and their clinical relevance. For the analysis of ALI, these covariates were: education level, poverty-to-income ratio, hypertension, diabetes, alcohol consumption, smoking status, physical activity level (PAmet), and sleep disorders. For the analysis of depressive symptoms, the model was identically adjusted with the addition of BMI. The results of these models were presented as hazard ratios (HRs) with their corresponding 95% confidence intervals (CIs).

To assess the joint effects, participants were stratified into groups based on their ALI and depressive symptom levels. We then utilized multivariable Cox regression, adjusting for the full set of covariates as specified in Model 3, to evaluate the mortality risk across these combined groups.

To flexibly model the dose-response relationship, we incorporated restricted cubic splines (RCS) into the fully adjusted Cox proportional hazards model (Model 3). This allowed us to visualize both linear and nonlinear associations between continuous ALI or PHQ-9 scores and mortality. A threshold analysis was subsequently performed to further explore the nature of these relationships. Furthermore, subgroup analyses stratified by key covariates were conducted to assess the robustness of our findings and explore potential effect modification. Finally, causal mediation analysis was conducted using a bootstrapping approach (5,000 resamples) to investigate the extent to which ALI and PHQ-9 scores mediate the association between osteoporosis and mortality outcomes. The analysis decomposed the total effect into direct and indirect effects, with 95% confidence intervals calculated using the bias-corrected and accelerated (BCa) bootstrap method. The proportion mediated was estimated as the ratio of the indirect effect to the total effect. All mediation analyses were adjusted for the same covariates used in the primary analyses.

All statistical tests were two-sided, with statistical significance set at P < 0.05. Data analysis for this study was conducted using IBM SPSS Statistics 25.0 and R version 4.4.1.

## Results

**Fig 1:**
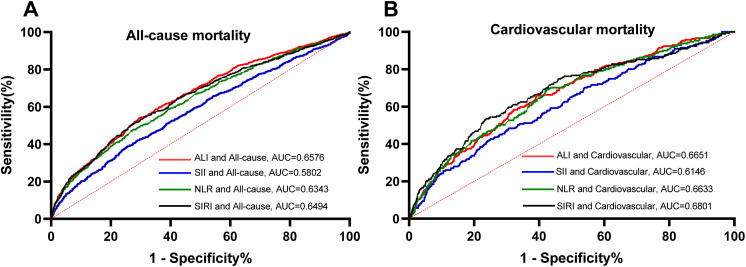
The time-dependent ROC of inflammation and nutrition-relative indicators for diagnosing overall survival in osteoporosis and osteopenia patients. Abbreviations: ALI, advanced lung cancer inflammation index; SII, systemic immune-inflammation index; NLR, neutrophil-to-lymphocyte ratio; SIRI, systemic inflammatory response index.

Between 2007 and 2023, the National Health and Nutrition Examination Survey (NHANES) included a total of 78,538 participants. After excluding individuals who did not meet the inclusion criteria or lacked essential data, the final study cohort consisted of 3,405 individuals diagnosed with osteoporosis and osteopenia, and 15,488 healthy controls (S1 Fig in S1 File). The mean age of the participants was 63 ± 12 years, with females comprising 50.81% and males comprising 49.19%. Table 1 presents the baseline characteristics of each group. In the analysis stratified by ALI three equal parts, significant differences were observed among the groups in variables such as gender, age, race, poverty-to-income ratio, and BMI (p < 0.05). Over the 16-year follow-up period, there were 862 cases of all-cause mortality and 211 cases of cardiovascular-related mortality. Furthermore, we assessed the differences in mortality prediction for patients with osteoporosis and osteopenia using ALI and common inflammatory markers through ROC curve analysis. As shown in Fig 1, ALI demonstrated superior predictive performance for both all-cause mortality and cardiovascular mortality, providing a comprehensive reflection of metabolic status.

**Table 1:** Baseline characteristics of the study cohort.

Study variables	Total (n = 3405)	No. of participants by ALI			P value
Q1 < 6.11 (n = 1125)	Q2：6.11–6.52 (n = 1124)	Q3: > 6.52 (n = 1156)
Age,years	62.74 ± 12.00	65.48 ± 12.50	62.12 ± 11.96	60.62 ± 10.98	<0.001
Sex,n(%)					<0.001
Male	1675 (49.19%)	639 (56.30%)	539 (47.49%)	497 (43.79%)	
Female	1730 (50.81%)	496 (43.70%)	596 (52.51%)	638 (56.21%)	
Race					<0.001
Mexican	563 (16.53%)	127 (11.19%)	223 (19.65%)	213 (18.77%)	
Hispanic	359 (10.54%)	98 (8.63%)	115 (10.13%)	146 (12.86%)	
Non-Hispanic white	1983 (58.24%)	758 (66.78%)	676 (59.56%)	549 (48.37%)	
Non-Hispanic black	360 (10.57%)	90 (7.93%)	84 (7.40%)	186 (16.39%)	
Other/multiracial	140 (4.11%)	62 (5.46%)	37 (3.26%)	41 (3.61%)	
Education level, n (%)					0.097
Never attended high school	1068 (31.37%)	330 (29.07%)	361 (31.81%)	377 (33.22%)	
High school and above	2337 (68.63%)	805 (70.93%)	774 (68.19%)	758 (66.78%)	
Poverty-to-income ratio, n (%)					0.01
Poor (≤1)	620 (18.21%)	185 (16.30%)	197 (17.36%)	238 (20.97%)	
Not poor (>1)	2785 (81.79%)	950 (83.70%)	938 (82.64%)	897 (79.03%)	
Smoking status, n (%)					0.051
Never	72 (2.11%)	13 (1.15%)	26 (2.29%)	33 (2.91%)	
Former	2125 (62.41%)	706 (62.20%)	716 (63.08%)	703 (61.94%)	
Current smoker	1208 (35.48%)	416 (36.65%)	393 (34.63%)	399 (35.15%)	
Alcohol use, n (%)					0.536
Never	864 (25.37%)	309 (27.22%)	272 (23.96%)	283 (24.93%)	
Mild	530 (15.57%)	180 (15.86%)	169 (14.89%)	181 (15.95%)	
Moderate	1673 (49.13%)	541 (47.67%)	578 (50.93%)	554 (48.81%)	
Heavy	338 (9.93%)	105 (9.25%)	116 (10.22%)	117 (10.31%)	
Hypertension, n (%)	1564 (45.93%)	538 (47.40%)	512 (45.11%)	514 (45.29%)	0.476
Diabetes mellitus, n (%)	551 (16.18%)	175 (15.42%)	187 (16.48%)	189 (16.65%)	0.689
Physical activity level, n (%)					0.15
Met PA (≤600)	1657 (48.66%)	579 (51.01%)	537 (47.31%)	541 (47.67%)	
Met PA (≥600)	1748 (51.34%)	556 (48.99%)	598 (52.69%)	594 (52.33%)	
BMI, kg/m2	27.38 ± 5.06	25.33 ± 4.48	27.53 ± 4.78	29.29 ± 5.11	<0.001
Sleep disorders	937 (27.52%)	303 (26.70%)	324 (28.55%)	310 (27.31%)	0.603
PHQ-9 score (%)					0.552
0-4	2581 (75.80%)	870 (76.65%)	854 (75.24%)	857 (75.51%)	
5-9	525 (15.42%)	165 (14.54%)	173 (15.24%)	187 (16.48%)	
≥ 10	299 (8.78%)	100 (8.81%)	108 (9.52%)	91 (8.02%)	

Abbreviations: ALI: advanced lung cancer inflammation index; BMI: body mass index; PHQ-9 score, Patient Health Questionnaire-9.

The proportional hazards regression analysis examined the intricate relationship between ALI, depressive symptoms, and mortality. In our fully adjusted model (Model 3), when ALI was treated as a continuous variable, the results demonstrated that ALI was negatively associated with both all-cause mortality and cardiovascular mortality, with HRs of 0.52 (95% CI: 0.45, 0.59) and 0.46 (95% CI: 0.36, 0.59), respectively. In comparison to individuals with low ALI levels, those with high ALI levels exhibited HRs of 0.44 (95% CI: 0.37, 0.53) for all-cause mortality and 1.06 (95% CI: 0.58, 1.94) for cardiovascular mortality. Additionally, patients with a PHQ-9 score ≥10 demonstrated a higher risk of both all-cause mortality (HR: 1.64, 95% CI: 1.28, 2.10) and cardiovascular mortality (HR: 2.06, 95% CI: 1.26, 3.35) compared to those with a PHQ-9 score between 0 and 4 (Table 2).

**Table 2:** HRs (95% CIs) for all-cause mortality and cardiovascular mortality derived from ALI and PHQ-9 scores for U.S. patients aged 40 years and older with osteoporosis or osteopenia in NHANES (2007-2023).

		Model1		Model2		Model3	
HR (95% CI)	p value	HR (95% CI)	p value	HR (95% CI)	p value
**All-cause mortality**							
**ALI**							
Continuous data		0.37(0.33,0.42)	<0.0001	0.53(0.47,0.60)	<0.0001	0.52(0.45,0.59)	<0.0001
Quartiles	Q1	Reference		Reference		Reference	
	Q2	0.52(0.45,0.61)	<0.0001	0.68(0.59,0.80)	<0.0001	0.67(0.58,0.79)	<0.0001
	Q3	0.32(0.26,0.38)	<0.0001	0.48(0.39,0.57)	<0.0001	0.44(0.37,0.53)	<0.0001
**PHQ-9 score**							
0-4		Reference		Reference		Reference	
5-9		1.15(0.96,1.38)	0.1309	1.45(1.21,1.74)	<0.0001	1.33(1.11,1.60)	0.0025
≥ 10		1.11(0.88,1.41)	0.3564	2.04(1.62,2.59)	<0.0001	1.64(1.28,2.10)	<0.0001
**Cardiovascular mortality**							
**ALI**							
Continuous data		0.30(0.24,0.38)	<0.0001	0.48(0.37,0.62)	<0.0001	0.46(0.36,0.59)	<0.0001
Quartiles	Q1	Reference		Reference		Reference	
	Q2	1.10(0.61,1.98)	0.7457	1.13(0.62,2.04)	0.6943	0.97(0.53,1.77)	0.9187
	Q3	1.39(0.80,2.43)	0.2481	1.36(0.76,2.41)	0.299	1.06(0.58,1.94)	0.853
**PHQ-9 score**							
0-4		Reference		Reference		Reference	
5-9		1.23(0.86,1.76)	0.2578	1.62(1.13,2.33)	0.0087	1.53(1.06,2.21)	0.0247
≥ 10		1.20(0.76,1.90)	0.4248	2.53(1.59,4.02)	<0.0001	2.06(1.26,3.35)	0.0038

Model 1: we did not adjust other covariant.

Model 2: we adjusted age, sex and race.

Model 3 on ALI: we adjusted sex, age, race, education, poverty-to-income ratio, hypertension, diabetes, alcohol use, smoking, PAmet and Sleep disorders.

Model 3 on Depression: we adjusted sex, age, race, education, poverty-to-income ratio, hypertension, diabetes, alcohol use, smoking PAmet, BMI and Sleep disorders.

In the joint analysis, a higher ALI level combined with a lower PHQ-9 score was significantly inversely associated with the risks of both all-cause mortality and cardiovascular mortality (Table 3). After full covariate adjustment, a combination of high ALI level and a lower PHQ-9 score was most protective. Specifically, compared to survivors with a PHQ-9 score ≥10 and low ALI levels, those with a PHQ-9 score <10 and high ALI levels exhibited a significantly lower risk of all-cause mortality.

**Table 3:** Joint association of ALI and PHQ-9 scores with all-cause and cardiovascular mortality among US osteoporosis or osteopenia age 40 years or older, NHANES, 2007 to 2023.

		Model1		Model2		Model3	
Mortality outcome	ALI	HR (95% CI)	p value	HR (95% CI)	p value	HR (95% CI)	p value
All cause							
PHQ-9 score ≥ 10	Low	Reference		Reference		Reference	
	Intermediate	0.51(0.31,0.84)	0.0088	0.60(0.36,1.00)	0.0522	0.53(0.31,0.88)	0.0138
	High	0.41(0.23,0.72)	0.0019	0.56(0.31,0.99)	0.046	0.52(0.39,0.92)	0.0257
PHQ-9 score < 10	Low	0.98(0.71,1.36)	0.9012	0.53(0.38,0.74)	0.0002	0.65(0.47,0.92)	0.0138
	Intermediate	0.52(0.37,0.73)	0.0001	0.37(0.27,0.52)	<0.0001	0.46(0.32,0.65)	<0.0001
	High	0.30(0.21,0.43)	<0.0001	0.25(0.18,0.36)	<0.0001	0.29(0.20,0.41)	<0.0001
Cardiovascular							
PHQ-9 score ≥ 10	Low	Reference		Reference		Reference	
	Intermediate	0.26(0.08,0.80)	0.0186	0.34(0.11,1.05)	0.0612	0.27(0.09,0.86)	0.026
	High	0.30(0.10,0.90)	0.0334	0.54(0.17,1.67)	0.2814	0.52(0.17,1.62)	0.2595
PHQ-9 score < 10	Low	0.80(0.45,1.43)	0.46	0.40(0.22,0.72)	0.002	0.48(0.26,0.88)	0.0168
	Intermediate	0.36(0.20,0.66)	0.001	0.25(0.14,0.46)	<0.0001	0.30(0.16,0.56)	0.0002
	High	0.19(0.10,0.37)	<0.0001	0.17(0.09,0.33)	<0.0001	0.19(0.10,0.37)	<0.0001

Model 1: we did not adjust other covariant.

Model 2: we adjusted age, sex and race.

Model 3: we adjusted sex, age, race, education, poverty-to-income ratio, hypertension, diabetes, alcohol use, smoking, PAmet and Sleep disorders.

**Fig 2:**
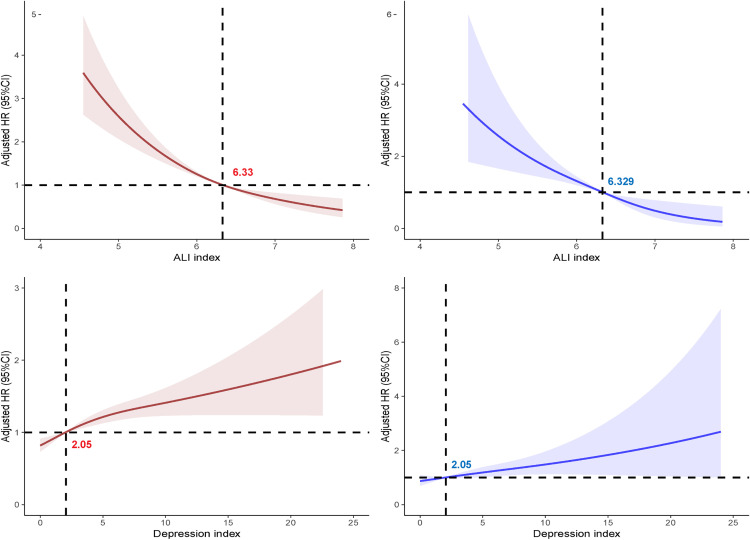
Association between ALI and Depression index with all-cause mortality (A, C) and cardiovascular mortality (B, D) in participants with OP. Adjusted for sex, age, race, education, poverty-to-income ratio, hypertension, diabetes, alcohol use, smoking, physical activity level and sleep disorders or BMI. The shaded areas represent the 95% CI.

As illustrated in Fig 2; After adjustment for all potential confounders as specified in Model 3, the RCS analysis demonstrated a negative correlation between ALI and both all-cause and cardiovascular mortality. Specifically, as ALI increased, HR for all-cause mortality exhibited a significant decrease. In contrast, higher PHQ-9 scores were positively correlated with both all-cause and cardiovascular mortality. To further elucidate the nature of these associations, a threshold analysis was conducted, revealing that ALI had a linear relationship with both all-cause and cardiovascular mortality, while PHQ-9 scores displayed a nonlinear relationship with these outcomes (refer to Table 4).

**Table 4:** Threshold analysis of ALI index and PHQ-9 scores on all cause and cardiovascular mortality in osteoporosis or osteopenia patients.

		Adjusted HR (95% CI)	P value	P for Log-likelihood ratio†
All-cause mortality				
ALI	Fitting by the standard linear model	0.52(0.46-0.59)	<0.0001	
	Inflection point:6.33			0.564
	Fitting by the two-piecewise linear model			
	ALI index<6.33	0.50(0.42-0.60)	<0.0001	
	ALI index>6.33	0.57(0.41-0.78)	0.0007	
Cardiovascular mortality				
ALI	Fitting by the standard linear model	0.46(0.36-0.59)	<0.0001	
	Inflection point:6.329			0.444
	Fitting by the two-piecewise linear model			
	ALI index<6.329	0.50(0.36-0.71)	<0.0001	
	ALI index>6.329	0.35(0.16-0.77)	0.0087	
All-cause mortality				
Depression	Fitting by the standard linear model	1.04(1.03-1.06)	<0.0001	
	Inflection point:7			0.027
	Fitting by the two-piecewise linear model			
	ALI index<7	1.08(1.04-1.11)	<0.0001	
	ALI index>7	1.01(0.98-1.05)	0.4958	
Cardiovascular mortality				
Depression	Fitting by the standard linear model	1.05(1.02-1.09)	0.0049	
	Inflection point:11			0.022
	Fitting by the two-piecewise linear model			
	ALI index<11	1.09(1.04-1.14)	0.0003	
	ALI index>11	0.90(0.77-1.06)	0.2074	

†Loglikelihood ratio is used to assess whether there is a statistical difference between two segmented linear models.

**Fig 3:**
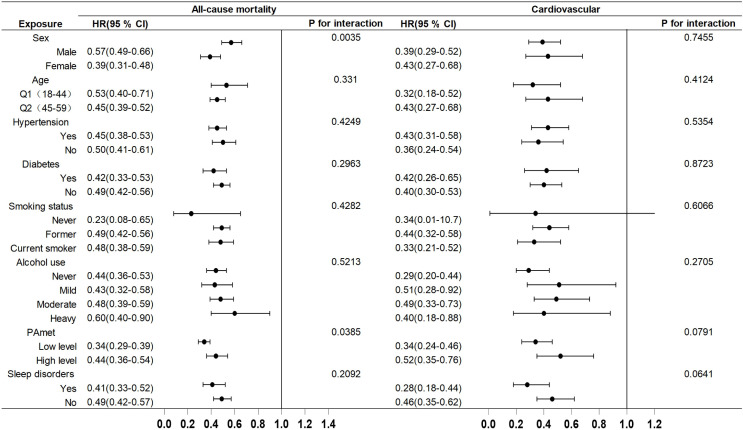
Subgroup analysis of the associations between ALI and all-cause and cardiovascular mortality. Adjusted for sex, age, race, education, poverty-to-income ratio, hypertension, diabetes, alcohol use, smoking, physical activity level and sleep disorders.

**Fig 4:**
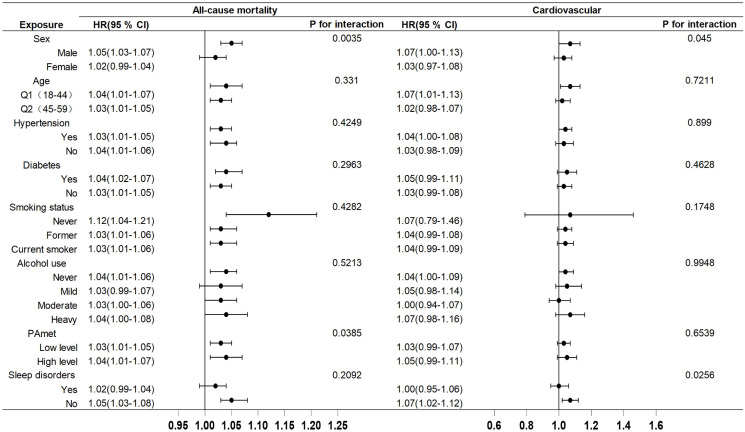
Subgroup analysis of the associations between PHQ-9 scores and all-cause and cardiovascular mortality. Adjusted for sex, age, race, education, poverty-to-income ratio, hypertension, diabetes, alcohol use, smoking, BMI, physical activity level and sleep disorders.

Additionally, a subgroup analysis was performed to examine the influence of various covariates on the relationship between ALI, PHQ-9 scores, and mortality (see Figs 3 and 4). The fully adjusted models revealed some potential effect modifications. In the stratified analysis of all-cause mortality, ALI exhibited a more protective effect in females than in males. Regarding the impact of exercise intensity, low-intensity exercise had a more pronounced protective effect on all-cause mortality, whereas PHQ-9 scores demonstrated a more detrimental impact on mortality in male patients. The relationship between exercise intensity and PHQ-9 scores was similar. Interestingly, patients without sleep disorders exhibited a stronger adverse effect of PHQ-9 scores on cardiovascular mortality.

Finally, causal mediation analysis with bootstrapping revealed that ALI mediated 3.9% of the association between osteoporosis and all-cause mortality, and 5.6% of the relationship between osteoporosis and cardiovascular mortality. Furthermore, PHQ-9 scores mediated 6.6% of the effect on cardiovascular mortality (S5 Table in S1 File). These findings suggest potential pathways through which osteoporosis may influence mortality outcomes, though causal inference is limited by the observational nature of the study.

## Discussion

In this study, we performed a retrospective cohort analysis using a nationally representative sample of osteoporosis patients to investigate the impact of inflammation and nutrition (ALI), as well as depressive symptoms (PHQ-9), on mortality. The findings provide the first evidence of the independent effects of inflammation, nutritional status, and depressive symptoms on mortality. Furthermore, the study demonstrates that individuals with higher ALI levels and no depressive symptoms exhibit a lower risk of both all-cause and cardiovascular mortality compared to those with lower ALI levels and/or more severe depressive symptoms.

The pathophysiology of osteoporosis is now understood to be critically influenced by an interplay of systemic factors, including the patient’s nutritional state, the presence of chronic inflammation, and persistent immune system activity [26–28]. Additionally, inflammatory responses and nutritional status are intricately linked to depressive symptoms in individuals with osteoporosis [11,29]. The primary finding of this study is that inflammation, nutritional status, and depressive symptoms are independently and synergistically associated with the mortality of osteoporosis patients. Existing studies, primarily utilizing ALI or PHQ-9 scores, have predominantly focused on the relationship between individual factors and the onset of osteoporosis [30,31]. In contrast, this study offers a more holistic perspective on mortality risk by simultaneously analyzing the prognosis of osteoporosis patients.

Patients with osteoporosis, particularly those in older age groups, are vulnerable to a range of comorbid conditions, including malnutrition, immune deficiency, inflammation, and metabolic disorders. Consequently, identifying composite indicators that reflect both nutritional status and inflammatory processes is significant [32–34]. Unlike traditional inflammatory markers, the ALI score incorporates both nutritional and inflammatory factors, offering a more holistic evaluation of a patient’s overall health. The ALI is primarily composed of neutrophil and lymphocyte counts, albumin levels, and BMI, all of which are closely associated with the onset and progression of osteoporosis [35–38]. Therefore, the ALI may prove to be a more reliable predictor of osteoporosis prognosis than other conventional indicators, as evidenced by AUC value of ALI in our analysis (Fig 2).

Furthermore, the results of our study suggest that, among the middle-aged and elderly population with osteoporosis, individuals exhibiting higher ALI levels experience significantly lower long-term mortality rates. This observation is consistent with prior studies that report a positive prognosis among various cancer survivors [39,40] and aligns with research demonstrating an independent association between ALI and the prevalence of OP in middle-aged and elderly patients with type 2 diabetes mellitus (T2DM) [31]. Additionally, our study identifies a significant nonlinear positive correlation between higher PHQ-9 scores and mortality in osteoporosis patients. This finding corroborates earlier studies that show a positive relationship between depression and osteoporosis, especially in elderly individuals or peri-menopausal women [30,41].

A notable proportion of osteoporosis patients also suffer from mental health disorders, including depression [12]. According to the Hispanic Elderly Epidemiological Study, 18% of elderly osteoporosis patients exhibit depressive symptoms [42], while a German nationwide health telephone survey reports that 14% of elderly osteoporosis patients show similar symptoms [43]. In our cohort, depressive symptoms in middle-aged and elderly osteoporosis patients were relatively mild, with approximately 8.8% of the participants scoring higher on the PHQ-9 scale. This rate is somewhat lower than the prevalence observed in other populations, which may be attributed to differences in measurement tools and variations in the average age of the populations studied. Furthermore, our study found that compared to osteoporosis and osteopenia patients with PHQ-9 scores of 0–4, those with scores ≥10 had a 64% and 106% increase in all-cause mortality and cardiovascular mortality, respectively. This finding aligns with previous research indicating that more severe depressive symptoms are typically associated with higher mortality risks [44]. In contrast to studies in the general population, our research provides novel and unique insights specifically within the context of osteoporosis.

The concept that systemic inflammation and depression synergistically increase mortality risk is well-established. Pioneering work by Lawes et al., for example, demonstrated that the combination of elevated C-reactive protein (CRP) and depressive symptoms was associated with a markedly higher risk of death in men [45]. This principle has since been extended to other clinical contexts and composite markers. For instance, studies in oncology have utilized the ALI, finding that cancer patients with a poor inflammatory-nutritional status (low ALI) combined with depression faced the worst prognoses, whereas those with a high ALI and good mental health had significantly better survival outcomes [46]. Building upon this foundational evidence, our study provides a more focused and arguably more striking analysis within the geriatric osteoporosis population. We specifically investigated the interplay between the ALI and depressive symptoms and found a profound impact on survival. Our results indicate that, compared to osteoporotic patients with depression and a low ALI score, those with a high ALI score and good mental health (PHQ-9: 0–4) experienced a 71% reduction in all-cause mortality and an even more pronounced 81% reduction in cardiovascular mortality. This finding is not merely confirmatory; it quantifies for the first time the substantial survival benefit of maintaining both a healthy inflammatory-nutritional state and good mental health in this vulnerable population, thereby addressing a significant gap in the literature.

The biological rationale for utilizing the ALI as a prognostic tool is strongly supported by the well-defined roles of its components in the pathophysiology of osteoporosis. NLR serves as a powerful index of the body’s net inflammatory state [47]. On one hand, neutrophils can actively contribute to bone degradation. Through the formation of neutrophil extracellular traps (NETs), they can induce localized tissue damage and promote the activity of osteoclasts, the primary cells responsible for bone resorption, thereby directly exacerbating bone loss [37]. On the other hand, lymphocytes play a crucial counter-regulatory role, helping to modulate and resolve the pro-inflammatory environment created by neutrophil activation [48]. Therefore, the NLR provides a holistic snapshot of this delicate balance between pro-inflammatory and regulatory immune functions. A higher NLR suggests a systemic shift towards inflammation, a state that is not only linked to the progression of osteoporosis itself but also to the increased risk of cardiovascular events, providing a clear mechanistic link between this biomarker and the adverse mortality outcomes observed in our cohort.

The link between poor nutritional status, as indicated by low serum albumin, and adverse skeletal health is well-documented [21]. Mechanistically, this association is partly attributable to albumin’s essential role in mineral homeostasis. As the primary transport protein for circulating calcium and phosphorus, insufficient albumin levels can compromise the systemic balance of these crucial bone-building minerals. This disruption in mineral availability is a direct contributor to reduced bone mineralization, ultimately leading to lower bone mineral density and an increased susceptibility to osteoporotic fractures [49].

As a key indicator of an individual’s nutritional status, Body Mass Index (BMI) shares a complex, non-linear relationship with skeletal health. A compelling body of research indicates that significant deviations from a normal BMI—encompassing both malnutrition (low BMI) and obesity (high BMI)—are established risk factors for osteoporosis [20]. The underlying biological mechanisms are twofold. On one hand, malnutrition directly compromises immune system function, impairing the body’s capacity to maintain skeletal homeostasis and repair microdamage. On the other hand, obesity impacts bone metabolism through more intricate pathways; it is not merely an issue of mechanical loading but is recognized as a chronic, low-grade inflammatory state that directly inhibits osteoblast function and promotes osteoclast activity through the release of various pro-inflammatory cytokines and the modulation of hormonal signals (e.g., the Wnt signaling pathway). Therefore, in the context of osteoporosis, BMI should be viewed as a window into the state of the “nutritional-inflammatory” axis. Maintaining an appropriate BMI is critical not only for ensuring adequate nutritional reserves but also for preserving immune and inflammatory system equilibrium, thereby mitigating detrimental effects on the skeleton. This perspective is highly consistent with our study’s central finding that the ALI, an index integrating both nutritional and inflammatory status, is a powerful predictor of patient mortality [50,51].

In conclusion, sustaining an appropriate BMI, normal serum albumin levels, and lower NLR levels may contribute to improved immune function, thereby enhancing prognosis. Moreover, poor physical and mental health may impact BMD and fracture risk via the hypothalamic-pituitary-adrenal (HPA) axis [52]. Future investigations should explore whether these associations are causal. If confirmed, these findings could offer a theoretical foundation for the development of intervention strategies aimed at improving treatment outcomes and overall well-being in middle-aged and elderly osteoporosis patients.

## Limitations

Although this study has yielded valuable insights, its limitations warrant careful consideration. Firstly, the study is largely based on cross-sectional laboratory data, which may restrict our capacity to capture longitudinal variations and responses to interventions. A more comprehensive understanding of the fluctuations in immune inflammation and nutritional status over time could be achieved through dynamic monitoring of ALI levels. Secondly, the assessment of depression is based on self-reported data from the PHQ-9 scale. While this scale has undergone rigorous validation by authoritative bodies such as the National Health Center, it is important to recognize the potential limitations and risks inherent in relying solely on self-reports, which may not fully or accurately reflect an individual’s depressive state. Thirdly, while our bootstrapping-based mediation analysis provides insights into potential pathways linking osteoporosis to mortality through ALI and PHQ-9 scores, the observational design limits causal inference. The identified mediating relationships should be interpreted as suggestive pathways rather than definitive causality. Cross-sectional data cannot establish temporal sequences, and unmeasured confounding may influence associations. Future longitudinal studies with repeated measurements and intervention studies are needed to validate these proposed pathways.

## Conclusions

Our study demonstrates a linear negative association between ALI and mortality risk in middle-aged and elderly osteoporosis patients, as well as a nonlinear positive relationship between PHQ-9 scores and mortality risk. Moreover, it underscores the importance of maintaining optimal nutrition and inflammation levels, alongside the absence of depression, in reducing the risk of both all-cause and cardiovascular mortality. These findings hold considerable significance for public health, particularly in the context of treating and preventing inflammation and depression in middle-aged and elderly individuals with osteoporosis. They offer valuable insights for clinicians in formulating more precise prevention and treatment strategies, ultimately contributing to improved patient prognosis.

## Supporting information

S1 File S1 Fig. Selection process for study cohorts. S1 Table. Covariate-specific information. S2 Table. Mediation analysis of the association between OP and the risk of all cause and cardiovascular mortality mediated by ALI and PHQ-9 scores.
(DOCX)

## Abbreviations

OP: Osteoporosis
NHANES: National Health and Nutrition Examination Survey
ALI: The advanced lung cancer inflammation index
NCHS: National Center for Health Statistics
DXA: Dual energy X-ray absorptiometry
IRB: Institutional Review Board
PHQ-9: The Patient Health Questionnaire-9
ICD-10: The International Classification of Diseases, 10th Edition
