# Supplementary material for: Combined effect of nutritional inflammation as well as depression on mortality in middle-aged and elderly people with osteoporosis and osteopenia
Source: PLoS One. 2025 Nov 3;20(11):e0335272. doi: 10.1371/journal.pone.0335272 (PMC12582478; doi:10.1371/journal.pone.0335272)
Supplement: S1 File — S1 Fig. Selection process for study cohorts. S1 Table. Covariate-specific information. S2 Table. Mediation analysis of the association between OP and the risk of all cause and cardiovascular mortality mediated by ALI and PHQ-9 scores. (DOCX) [file pone.0335272.s001.docx]

# **Appendix:**

**S1 Fig. Selection process for study cohorts.**

**
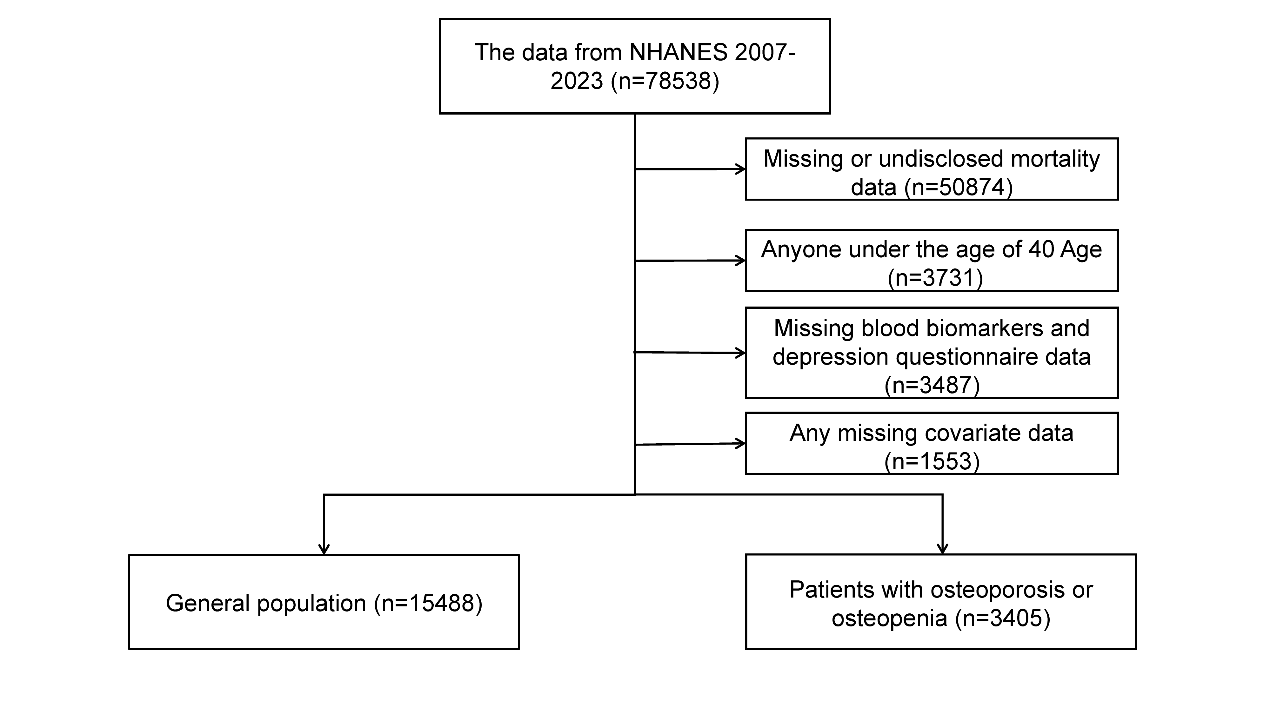
**

**S1 Table.** **Covariate-specific information.**

Smoking status was classified according to participants' responses to survey questions (SMQ020: "Have you ever smoked 100 cigarettes?" SMQ040: "What is your current smoking status?") to categorize individuals into never smokers, former smokers, and current smokers. Never smokers are defined as those who have never smoked 100 cigarettes in their lifetime and are not currently smoking. Current smokers are those who have smoked 100 or more cigarettes and are currently smoking. Former smokers are those who have smoked 100 or more cigarettes but have quit smoking. Alcohol consumption was similarly classified based on self-reported drinking frequency, with categories including heavy drinkers, moderate drinkers, light drinkers, and never drinkers. Heavy drinkers are defined as individuals consuming ≥4 or 5 drinks per day; moderate drinkers consume ≤3 drinks per day; light drinkers have consumed alcohol fewer than 12 times in the past year; and never drinkers are those who report never having consumed alcohol.

The diagnosis of diabetes and hypertension was confirmed through a combination of questionnaire responses and laboratory data to ensure precision. Relevant survey questions include: "Has a doctor ever told you that you have diabetes?" "Do you use insulin?" "Do you use oral hypoglycemic agents?" Diagnostic laboratory criteria for diabetes include fasting blood glucose ≥7.0 mmol/L, HbA1c ≥6.5%, and oral glucose tolerance test (OGTT) blood glucose ≥11.1 mmol/L. In a similar manner, hypertension diagnosis was based on multiple blood pressure readings ≥130/80 mmHg or a self-reported diagnosis of hypertension confirmed by a physician.

Different physical activities have distinct metabolic equivalent (MET) values, and NHANES provides recommended MET values for each type of exercise. The physical activity questionnaire (PAQ) includes categories such as vigorous work activity (MET=8), moderate work activity (MET=4), walking or cycling (MET=4), vigorous leisure-time physical activity (MET=8), and moderate leisure-time physical activity (MET=4). The participants' physical activity (PA) levels were calculated based on MET values, activity types, weekly frequency, and duration. The PA calculation formula is: PA (MET-h/week) = MET × weekly frequency × duration of each physical activity. Based on PA values, participants were categorized into low-intensity physical activity (≤600 MET-h/week) and high-intensity physical activity (>600 MET-h/week) groups.

Sleep disorder status was assessed using the SLQ060 and SLQ050 question modules in NHANES: "Has a doctor or other health professional ever told you that you have a sleep disorder?" and "Have you ever reported any sleep problems?" Individuals who responded "yes" were classified as having sleep disorders and included in the analysis. Furthermore, the SLQ070 question module includes self-reported symptoms of sleep disorders such as sleep apnea, insomnia, and restless leg syndrome. Individuals who answered "yes" to any of these symptoms were also considered to have sleep disorders.

|  | **Non-adjusted β (95%CI)P-value** | **Adjust I β (95%CI) P-value** | **Adjust II β(95%CI) P-value** |
| --- | --- | --- | --- |
| **ALI** | | | |
| All-cause mortality | | | |
| Direct effect | 0.093 (0.115, 0.138) <0.0001 | 0.064 (0.056, 0.072) <0.0001 | 0.093 (0.082, 0.107) <0.0001 |
| Indirect effect | 0.004 (0.011, 0.015) <0.0001 | 0.003 (0.003, 0.004) <0.0001 | 0.004 (0.003, 0.005) <0.0001 |
| Total effect | 0.097 (0.128, 0.151) <0.0001 | 0.067 (0.059, 0.076) <0.0001 | 0.097 (0.086, 0.111) <0.0001 |
| PM, % | 3.9 | 5.1 | 3.9 |
| P-value | <0.0001 | <0.0001 | <0.0001 |
| Cardiovascular mortality | | | |
| Direct effect | 0.028 (0.023, 0.035) <0.0001 | 0.012 (0.008, 0.017) <0.0001 | 0.021 (0.014, 0.029) <0.0001 |
| Indirect effect | 0.004 (0.003, 0.005) <0.0001 | 0.001 (0.0007, 0.001) <0.0001 | 0.001 (0.001, 0.002) <0.0001 |
| Total effect | 0.032 (0.026, 0.039) <0.0001 | 0.013 (0.009, 0.018) <0.0001 | 0.022 (0.016, 0.030) <0.0001 |
| PM, % | 12.5 | 7.4 | 5.6 |
| P-value | <0.0001 | <0.0001 | <0.0001 |
| **Depression** | | | |
| All-cause mortality | | | |
| Direct effect | 0.126 (0.115, 0.138) <0.0001 | 0.064 (0.056, 0.072) <0.0001 | 0.098 (0.085, 0.111) <0.0001 |
| Indirect effect | 0.013 (0.011, 0.015) <0.0001 | 0.003 (0.003, 0.004) <0.0001 | 0.0005 (-0.0001, 0.001) 0.118 |
| Total effect | 0.139 (0.128, 0.151) <0.0001 | 0.067 (0.059, 0.076) <0.0001 | 0.098 (0.086, 0.111) <0.0001 |
| PM, % | 9.2 | 5.1 | 0.54 |
| P-value | <0.0001 | <0.0001 | 0.118 |
| Cardiovascular mortality | | | |
| Direct effect | 0.028 (0.023, 0.035) <0.0001 | 0.012 (0.008, 0.017) <0.0001 | 0.012 (0.008, 0.016) <0.0001 |
| Indirect effect | 0.004 (0.003, 0.005) <0.0001 | 0.001 (0.0007, 0.001) <0.0001 | 0.001 (0.0005, 0.001) <0.0001 |
| Total effect | 0.032 (0.026, 0.039) <0.0001 | 0.013 (0.009, 0.018) <0.0001 | 0.013 (0.009, 0.017) <0.0001 |
| PM, % | 12.5 | 7.4 | 6.6 |
| P-value | <0.0001 | <0.0001 | <0.0001 |

**S2 Table. Mediation analysis of the association between OP and the risk of all cause and cardiovascular mortality mediated by ALI and PHQ-9 scores.**

Crude model: we did not adjust other covariant.

Model I: we adjusted age, sex and race.

Model II on ALI: we adjusted sex, age, race, education, poverty-to-income ratio, hypertension, diabetes, alcohol use, smoking, PAmet and Sleep disorders.

Model II on Depression: we adjusted sex, age, race, education, poverty-to-income ratio, hypertension, diabetes, alcohol use, smoking PAmet,BMI and Sleep disorders.
